# Adiponectin/AdipoR1 Axis Promotes IL-10 Release by Human Regulatory T Cells

**DOI:** 10.3389/fimmu.2021.677550

**Published:** 2021-05-18

**Authors:** Patricia Ramos-Ramírez, Carina Malmhäll, Omar Tliba, Madeleine Rådinger, Apostolos Bossios

**Affiliations:** ^1^ Krefting Research Centre, Department of Internal Medicine and Clinical Nutrition, Institute of Medicine, Sahlgrenska Academy, University of Gothenburg, Gothenburg, Sweden; ^2^ Department of Biomedical Sciences, College of Veterinary Medicine, Long Island University, Brookville, NY, United States; ^3^ Department of Respiratory Medicine and Allergy, Karolinska University Hospital, Huddinge and Department of Medicine, Huddinge, Karolinska Institutet, Stockholm, Sweden

**Keywords:** adiponectin receptor 1, regulatory T cells, interleukin-10, adiponectin, type 2 inflammation

## Abstract

**Background:**

Adiponectin is an important immunomodulatory mediator in inflammatory conditions. While we previously showed that adiponectin receptor 1 (AdipoR1) is expressed in murine regulatory T cells (Tregs), its expression in human Tregs remain unknown. Here, we examined the expression of AdipoR1 in human Tregs and whether its ligand, globular adiponectin (gAd) affects the Treg ability to secrete IL-10 and the role of Type 2 (T2) inflammation in such process.

**Methods:**

Human Tregs from peripheral blood were analyzed by flow cytometry for AdipoR1, Helios and IL-10 expression. CD4^+^ T cells enriched from peripheral blood mononuclear cells (PBMCs) were cultured in the presence or the absence of gAd or the chemical adiponectin receptor agonist, AdipoRon, or in a T2 cytokine milieu. Flow cytometry was then used to assess intracellular IL-10, IL-10 secreting cells, FOXP3 and Helios expression, and phosphorylated p38 MAP kinase (MAPK). IL-10 levels in CD4^+^ T cell supernatants were quantified by ELISA.

**Results:**

We found that a subset of human Tregs expressed AdipoR1. Importantly, more Helios^-^ cells expressed AdipoR1 than Helios^+^ cells. Likewise, there was a higher frequency of IL-10^+^ cells within Helios^-^ AdipoR1^+^ Tregs compared to Helios^+^ AdipoR1^+^ Tregs. In contrast, the IL-10 mean fluorescence intensity (MFI) was higher in Helios^+^ AdipoR1^+^ Tregs compared to Helios^-^AdipoR1^+^ Tregs. When human CD4^+^ T cells were treated with gAd or AdipoRon, a significant increase in IL-10 secretion, FOXP3 expression, and p38 MAPK phosphorylation was observed in Helios^-^ AdipoR1^+^ Tregs. Interestingly, gAd under T2 cytokine milieu significantly increased the intracellular levels of IL-10, mainly in Helios^+^ AdipoR1^+^ Tregs, and IL-10 levels in supernatants of CD4^+^ T cells.

**Conclusions:**

Collectively, our findings suggest that adiponectin/AdipoR1 axis promotes IL-10 release by Tregs, mainly in Helios^-^ Tregs, and the effect was amplified by T2 inflammation in Helios^+^ Tregs.

## Introduction

Regulatory T cells (Tregs) are critical modulators of immune responses and play an important role in maintaining peripheral tolerance (1, 2). Tregs express FOXP3, a key transcription factor for their development and function (2, 3). According to their origin, FOXP3^+^ Tregs can be divided into two major subsets, i) thymus-derived Tregs (tTregs) and ii) peripherally-induced Tregs (pTregs) (3–5). Although these cell populations are considered phenotypically undistinguishable, several studies suggested that Helios, an Ikaros family transcription factor, could discriminate between tTregs and pTregs (6–9). Tregs are capable to further differentiate in response to specific inflammatory signals where they can express diverse markers associated with specific functional features or tissue localization (10–13). Interestingly, FOXP3^+^ Tregs constitute more than half of the CD4^+^ T cells in the abdominal fat of lean mice and have been implicated in controlling insulin resistance through IL-10 induction (14). We previously showed that the majority of adipose tissue-resident Tregs in lean mice were Helios^+^ FOXP3^+^ Tregs and expressed higher levels of adiponectin receptor 1 (AdipoR1) than their counterpart in the spleen (15). However, AdipoR1 expression and its function in human Tregs remain unknown.

Adiponectin is the most abundant adipocyte-derived protein in human plasma, with circulating concentrations that oscillate from 5 to 30 µg/ml; it represents 0.01% of the total plasma proteins (16–18). While adiponectin levels are inversely correlated with obesity and insulin resistance, weight loss and exercise induce adiponectin synthesis (19–21). Circulating adiponectin exists as a full-length protein that forms oligomeric complexes and also as a globular C-terminal fragment (gAd) generated by proteolytic cleavage (22, 23). These adiponectin isoforms have distinct biological properties and affinities for the adiponectin receptors (AdipoRs). While AdipoR1 is a high-affinity receptor for gAd, AdipoR2 binds preferentially to the full-length protein (24–26). The full-length adiponectin forms high molecular multimers and binds to T-cadherin, which is critical for cell adhesion and energy homeostasis (16, 27).

Adiponectin has a broad range of biological functions including effects on the metabolism, immune responses and inflammation (28, 29). For instance, adiponectin attenuates the adhesion and the chemotaxis of human eosinophils (30) and reduces type 2 (T2) cytokine levels in a mouse model of allergic airway inflammation (31). In human dendritic cells and macrophages, adiponectin induces the synthesis of anti-inflammatory cytokines such as IL-10 and IL-1RA (32, 33). Importantly, while adiponectin-treated dendritic cells promote Tregs expansion (34), the direct effect of adiponectin on the functions of Tregs has not been demonstrated. Hence, here we examined whether human circulating Tregs express the AdipoR1 and whether human Tregs produce IL-10 in response to adiponectin. Moreover, we also studied whether T2 inflammation affects AdipoR1^+^ Tregs response to adiponectin.

## Materials and Methods

### Samples

This study included human peripheral whole blood samples and fresh buffy coats. Peripheral blood was collected in EDTA Vacutainer^®^ tubes (Becton Dickinson, USA) from healthy volunteers who gave their oral informed consents. The ethical approval was granted by the regional Ethical Approval Committee (no 593-08). The fresh buffy coats were obtained from healthy blood donors (Blood Donor Center, Sahlgrenska University Hospital, Gothenburg). All samples were completely anonymized.

### Phenotypic Analysis of Human Circulating Tregs

For *ex vivo* phenotypic analysis, peripheral blood was first diluted 1:1 in serum free RPMI 1640. Samples were then treated with phorbol 12-myristate 13-acetate (PMA, 50 ng/ml of cell suspension; Sigma-Aldrich, St Louis, MO, USA) and ionomycin (1 μg/ml of cell suspension, Sigma-Aldrich) for 4 h at 37°C. Brefeldin A (10 μg/ml of cell suspension; Sigma-Aldrich) was added for the last 3 h of incubation to inhibit protein transport before cells were stained with different surface markers (**Table 1**). Viable cells were determined by using the Live/Dead fixable Aqua stain kit (Life Technologies, Invitrogen™). Surface staining was followed by red blood cell lysis using BD FACS™ Lysing Solution (BD Pharmingen™ San Jose, CA, USA). Intracellular staining was performed using the Foxp3 Staining Buffer Set (eBioscience) according to the manufacturer’s instructions. A solution of human IgG (1 mg/ml; Sigma Aldrich) was added for at least 15 minutes at 4°C prior incubation with antibodies to prevent any non-specific binding. Antibodies used in this study are listed in **Table 1**. Samples were then processed on FACSVerse™ flow cytometer using FACS Suite software (BD Biosciences, San Jose, CA, USA) and data were analyzed using FlowJo Version 9.3.2 Software^®^ (Tri star Inc, Ashland, OR, USA). All the gates were determined using the fluorescence minus one (FMO) approach or matched isotype controls (AdipoR1, Helios and IL-10; **Table 1**). The gating strategy to identify Treg subsets was performed as follows: dead cells (positive for LD Aqua dye) were excluded and lymphocytes were identified using forward and side scatter properties (FSC-A *vs* SSC-A), cell doublets (FSC-A *vs* FSC-H) were excluded and CD4^+^ T cells were gated from single cells and analyzed for CD25 expression. Helios^+^ FOXP3^+^ and Helios^-^ FOXP3^+^ cells were gated on CD4^+^ CD25^+^ cells and then analyzed for AdipoR1 and IL-10 expression.

**Table 1:** Antibodies used for flow cytometry.

Antibody / Clone	Labelling	Manufacture	Catalogue no.
CD4/SK3	BV510	BD Biosciences	562970
CD4/RPA-T4	APC-H7	BD Biosciences	560158
CD251/M-A251	PE-Cy7	BD Biosciences	557741
AdipoR1	Unconjugated	Phoenix Pharmaceuticals	G-OO I -44
FOXP3 / 236A/E7	PE/PercP-Cy5.5	eBioscience I BD Bioscience	12-4777 / 561493
Helios / 227-6	PE 1 APC	eBioscience	12-9883/ 17-9883
IL-IO / JES3-9D7	BV421	BD Biosciences	564053
IL-IO / IL-IO Secretion Assay Kit	PE	Miltenyi Biotec	130-090-434
Phospho-p38 MARK/36/p38	PE-Cy7	BD Biosciences	560241
Donkey anti-rabbit lgG secondary Ab / Poly4064	F ITC	BioLegend	406403
Armenian Hamster lgG, Isotype control/eBio299Arm	PE/APC	eBiosciences	12-4888/ 17-4888
Rat lgG1 K, Isotype control / R3-34	BV421	BD Biosciences	562868

### Human PBMCs and CD4^+^ T Cells Isolation

Peripheral blood mononuclear cells (PBMCs) were obtained from fresh buffy coats and were isolated by density gradient centrifugation using Ficoll-Paque™ PLUS Media (GE Healthcare Bio-Sciences, Uppsala, Sweden). CD4^+^ T lymphocytes were enriched from PBMCs and purified by negative selection using magnetic separation according to the manufacturer´s protocol (Human CD4 T Lymphocyte Enrichment Set-DM; BD IMag™, BD Pharmingen™). The purity was evaluated by flow cytometry (>95%).

### Measurement of IL-10-Secreting Cells

CD4^+^ T cells were cultured in serum-free TexMACS™ medium (Miltenyi Biotec GmbH, Bergisch Gladbach, Germany). We used a supplemented serum-free medium in order to prevent the ability of additional factors to stimulate IL-10 production since endogenous adiponectin present in the fetal bovine serum has been shown to be physiologically active (35). Cells were treated with recombinant human (rh) gAd (10 μg/ml; R&D Systems^®^, Minneapolis, MN, USA) or a synthetic small-molecule agonist of AdipoRs, AdipoRon (8 μM/ml; Tocris Bioscience, UK) for 4 h or 16 h. As positive control for IL-10 production, cells were treated with PMA (50 ng/ml) and ionomycin (1 μg/ml) for 4 h. IL-10-secreting cells were assessed using the IL-10 Secretion Assay detection kit according to the manufacturer’s protocol (MACS; Miltenyi Biotec). Briefly, CD4^+^ T cells were labelled with IL-10 catch reagent and a secretion period of 45 minutes at 37°C was performed. Cells were then labelled with IL-10 detection antibody and counterstained for CD4, AdipoR1, FOXP3 and Helios or isotype controls (**Table 1**). Flow cytometry analysis of IL-10-secreting cells was performed as described above.

### Measurement of Intracellular IL-10 Levels

CD4^+^ T cells were cultured and treated as described above, with the exception of brefeldin A (10 μg/ml) that was added for the last 3 h of incubation. CD4^+^ T cells were harvested and the intracellular IL-10 levels were measured by flow cytometry.

### Cell Culture in T2 Inflammatory Milieu

CD4^+^ T cells were cultured in serum-free AIM V™ medium (Life Technologies Inc., Gaithersburg, MD) supplemented with rhIL-2 (400 U/ml; R&D Systems^®^). To address the stability of *ex vivo* isolated CD4^+^ T cells, cells were seeded in plates pre-coated with anti-CD3 and soluble anti-CD28 antibodies (1 μg each/ml; BD Pharmingen™, BD Biosciences). To obtain a T2 cytokine milieu, rhIL-4 (10 ng/ml), and anti-human (h) IL-12 (10 μg/ml) and anti-hIFN-γ (10 μg/ml) neutralizing antibodies (all from BD Pharmingen™) were added as previously described (36). Cells were cultured for 5 days in the presence or absence of gAd (10 μg/ml). After the treatment, cells were re-stimulated with PMA (10 ng/ml) and ionomycin (500 ng/ml) for 4 h and brefeldin A (10 μg/ml) was added in the last 3 h of the stimulation. Cells were harvested and washed with PBS, and intracellular staining was performed.

### Intracellular Staining and Flow Cytometric Analysis

CD4^+^ T cells were stained either with Live/Dead fixable Aqua stain kit (Invitrogen™) or 7AAD (BD Pharmingen™), and surface antibodies (**Table 1**). Staining for FOXP3, Helios, IL-10 and phospho-p38 MAPK (**Table 1**) was performed using the FOXP3 Staining Buffer Set (eBioscience) according to the manufacturer’s instructions. A solution of human IgG was added for at least 15 minutes at 4°C prior incubation with antibodies. Samples were analyzed on an FACSVerse™ flow cytometer using FACS Suite software (BD Biosciences, San Jose, CA, USA), and analyzed with FlowJo Version 9.3.2 Software^®^ (Tri star Inc, Ashland, OR, USA). All the gates were determined using the fluorescence minus one (FMO) approach or matched isotype controls (AdipoR1, Helios and IL-10; **Table 1**). Single CD4^+^ T cells were acquired through gating strategy mentioned above. The continuous expression of AdipoR1, IL-10, and phospho-p38 MAPK was performed on CD4^+^ FOXP3^+^ Helios^+^ or CD4^+^ FOXP3^+^ Helios^-^ cells. The gating strategy to identify Treg subsets is described in the Figure captions.

### Enzyme-Linked Immunosorbent Assay (ELISA)

Following incubation under T2 cytokine conditions, CD4^+^ T cells were harvested and centrifuged, and culture supernatants were collected and analyzed for IL-10 using ELISA DuoSet kit (R&D Systems^®^, catalogue no. DY217B-05) according to the manufacturer´s instructions. Absorbance was measured on a Varioskan™ LUX multimode microplate reader (Thermo Fisher Scientific). Of note, brefeldin A was not added to avoid inhibition of IL-10 secretion.

### Statistical Analysis

Data were analyzed with GraphPad Prism version 7.0b (GraphPad Software, La Jolla, CA, USA), and were expressed as means ± SEM. Data were tested for normal distribution by applying Shapiro-Wilk test. As variables were normally distributed, statistical analysis was performed using Student’s t-test or one-way ANOVA with Tukey’s multiple-comparisons *post-hoc* test. Pearson’s correlation analysis (*r_P,_*Pearson’s r correlation coefficient) was used to determinate the association between AdipoR1 and IL-10 expression. Statistical significance was defined as ^*^ *P < 0.05*, ^**^ *P < 0.01*, and ^***^ *P < 0.001*.

## Results

### Human Circulating FOXP3^+^ Tregs Express AdipoR1

**Figure 1:**
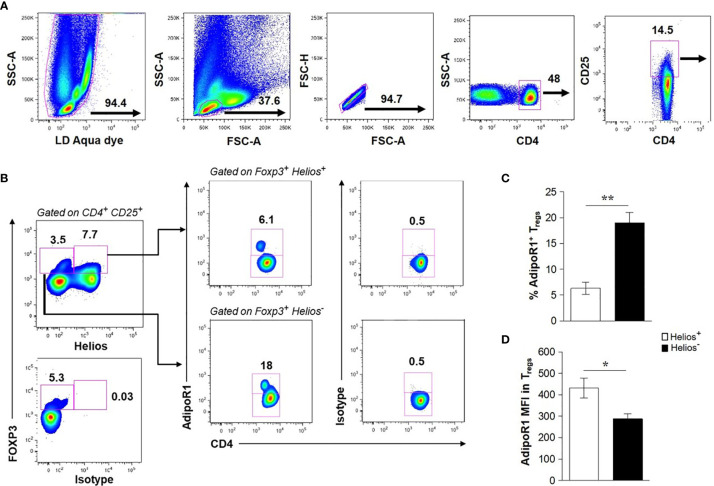
Human Circulating FOXP3^+^ Tregs Express AdipoR1. Flow cytometry analysis of AdipoR1 expression in Treg subsets was performed in peripheral whole blood from healthy donors. **(A)** Gating strategy of human circulating CD4^+^ CD25^+^ T cells. Dead cells, positive for LD Aqua dye, were excluded and lymphocytes were gated. CD4^+^ T cells were gated from single cells (excluding of doublets, FSC-A *vs* FSC-H) and analyzed for CD25 expression. **(B)** Helios^+^ and Helios^-^ FOXP3^+^ Tregs were gated on CD4^+^ CD25^+^ cells and then analyzed for AdipoR1 expression. FACS plots are from a representative experiment. **(C)** Frequency of AdipoR1^+^ cells and **(D)** MFI (mean fluorescence intensity) AdipoR1 in Helios^+^ and Helios^-^ FOXP3^+^ Tregs. Data were obtained from four independent experiments and are shown as the mean ± SEM of n=5. Student’s t-test, *P< 0.05 and **P< 0.01.

Human CD4^+^ T cells have been shown to preferentially express the AdipoR1 (37, 38); however, the expression of such receptor in Tregs has not been studied. Therefore, we sought to determine whether human circulating Tregs expressed AdipoR1. To this end, freshly collected whole blood cells were stained for two subpopulations of Tregs (CD4^+^ CD25^+^ FOXP3^+^ Helios^+^ or CD4^+^ CD25^+^ FOXP3^+^ Helios^-^) and analyzed by flow cytometry (**Figures 1A, B**). We found a lower frequency of AdipoR1 in Helios^+^ Tregs than that of Helios^-^ Tregs (6.3% ± 1.201 *vs* 19% ± 1.995, respectively, **Figure 1C**). In contrast, the intensity of AdipoR1 expression in Tregs, shown as the mean fluorescence intensity (MFI), was significantly higher in Helios^+^ Tregs than that of Helios^-^ Tregs (432 ± 46.22 *vs* 287.2 ± 24.47 respectively, **Figure 1D**).

### AdipoR1 Expression Correlates With IL-10 Production in FOXP3 Tregs

**Figure 2:**
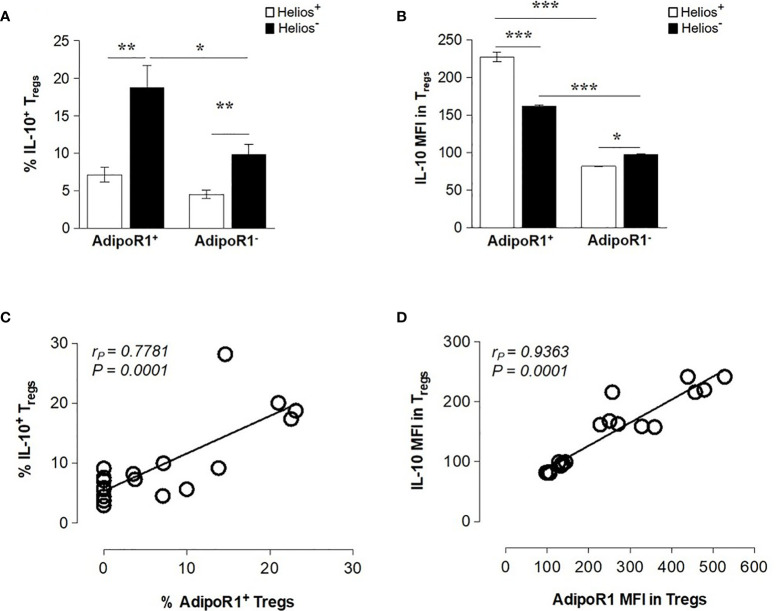
AdipoR1 Expression Correlates with IL-10 Production in FOXP3 Tregs. Human circulating AdipoR1^+^ and AdipoR1^-^ Treg subsets were analyzed by flow cytometry for IL-10 expression. **(A)** Frequency of IL-10^+^ cells and **(B)** MFI IL-10 in AdipoR1^+^ and AdipoR1^-^ Treg subsets (Helios^+^ and Helios^-^ cells). **(C)** The frequency or **(D)** MFI of AdipoR1 and IL-10 in Helios^+^ and Helios^-^ Tregs were plotted on a scatter plot and correlation analysis was performed. Cells were gated as shown in **Figure 1A** **(B)** Data are means ± SEM of n=5. Student’s t-test, *P< 0.05, **P< 0.01, and ***P< 0.001. Correlations were performed by using Pearson’s correlation coefficient (*r_P_*).

Previous studies have showed that Helios^-^ but not Helios^+^ human FOXP3 Tregs produce a variety of cytokines *ex vivo*, including the anti-inflammatory cytokine IL-10 (39, 40). Therefore, we next examined the ability of human AdipoR1^+^ and AdipoR1^-^ Treg subsets to produce IL-10. As shown in **Figure 2A**, Helios^-^ Tregs contained an increased frequency of IL-10^+^ cells as compared with Helios^+^ Tregs (both AdipoR1^+^ and AdipoR1^-^ cells). However, the frequency of IL-10^+^ cells was more prominent in Helios^-^ AdipoR1^+^ Tregs than in Helios^-^ AdipoR1^-^ Tregs. Strikingly, the intensity of IL-10 expression (MFI) was significantly higher in both Helios^+^ and Helios^-^ AdipoR1^+^ cells as compared with their counterparts within AdipoR1^-^ cells (**Figure 2B**). Moreover, Helios expression differentially affected the intensity of IL-10 expression in AdipoR1^+^ and AdipoR1^-^ populations. Indeed, while Helios^+^ AdipoR1^+^ Tregs expressed more IL-10 than Helios^-^ AdipoR1^+^ Tregs, Helios^+^ AdipoR1^-^ Tregs expressed less IL-10 than Helios^-^ AdipoR1^-^ Tregs (**Figure 2B**). Interestingly, AdipoR1 expression in Tregs showed a positive correlation with IL-10 production and expression (frequency, **Figure 2C**; MFI, **Figure 2D**). Taken together, these data suggest that Tregs expressing AdipoR1 produce more IL-10.

### gAd Induces IL-10 Secretion in Helios^-^ AdipoR1^+^ Tregs

**Figure 3:**
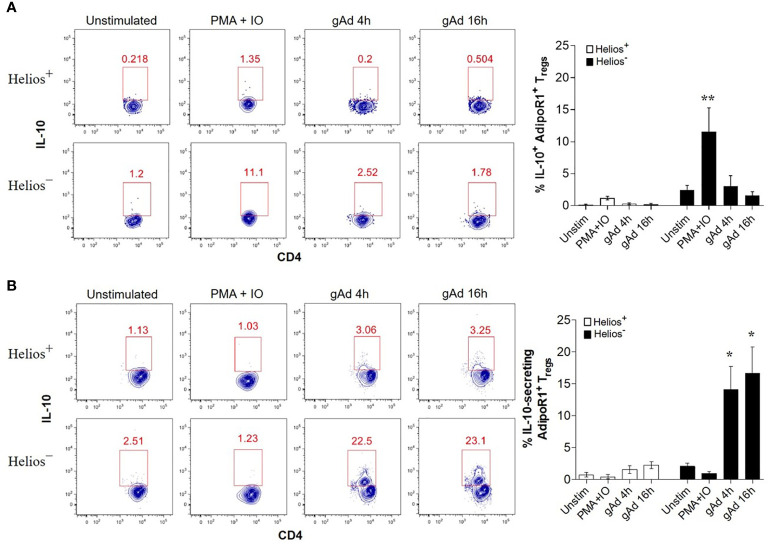
gAd induces IL-10 secretion in Helios^-^ AdipoR1+ Tregs. CD4^+^ T cells were cultured in the presence of gAd and then analyzed by flow cytometry. **(A)** Representative FACS plots show the intracellular levels of IL-10 in Helios^+^ AdipoR1^+^ Tregs (upper) and Helios^-^ AdipoR1^+^ Tregs (bottom). The graph (right) summarizes the frequencies of IL-10^+^ AdipoR1^+^ Treg subsets. **(B)** Representative FACS plots show the percentage of IL-10-secreting cells within Helios^+^ AdipoR1^+^ Tregs (upper) and Helios^-^ AdipoR1^+^ Tregs (bottom). The graph (right) summarizes the frequencies of IL-10-secreting cells within AdipoR1^+^ Treg subsets. Analysis of intracellular IL-10^+^ cells or IL-10-secreting cells was performed in CD4^+^ FOXP3^+^ Helios^+^ AdipoR1^+^ Tregs and in CD4^+^ FOXP3^+^ Helios^-^ AdipoR1^+^ Tregs. Data are means ± SEM of n=4-6 blood donors. One-way ANOVA with Tukey’s multiple comparisons test, *P< 0.05 and **P< 0.01.

Adiponectin is known to induce IL-10 production in a variety of immune cells (32, 33, 41, 42). Since AdipoR1 preferentially binds to gAd isoform (26), we next investigated whether gAd induces IL-10 expression in AdipoR1^+^ Tregs. To this end, CD4^+^ T cells enriched from buffy coats were treated with gAd for 4 h or 16 h and intracellular IL-10 was measured by flow cytometry. We found that while PMA/ionomycin significantly enhanced intracellular IL-10 expression in Helios^-^ AdipoR1^+^ Tregs, no effect was seen in Helios^+^ AdipoR1^+^ Tregs (**Figure 3A**). Strikingly, gAd treatment for 4 or 16 h did not affect the intracellular expression of IL-10 in AdipoR1^+^ Treg subsets (**Figure 3A**). Since gAd did not affect the intracellular levels of IL-10, we next sought to determine whether gAd induces the IL-10 secretion in AdipoR1^+^ Tregs. We found that PMA/ionomycin treatment did not increase the frequency of IL-10-secreting AdipoR1^+^ Tregs (**Figure 3B**). Strikingly, while gAd did not affect the frequency of IL-10-secreting cells in Helios^+^ AdipoR1^+^ Tregs, it significantly increases such frequency in Helios^-^ AdipoR1^+^ Tregs at both 4 and 16 h of treatment (**Figure 3B**). These results clearly indicate that gAd induces a rapid secretion of IL-10 by Helios^-^ AdipoR1^+^ Tregs.

### gAd Induces FOXP3 in CD4^+^ T Cells, p38 MAPK Activation in Helios^-^ Cells and AdipoR1 Expression in Tregs

**Figure 4:**
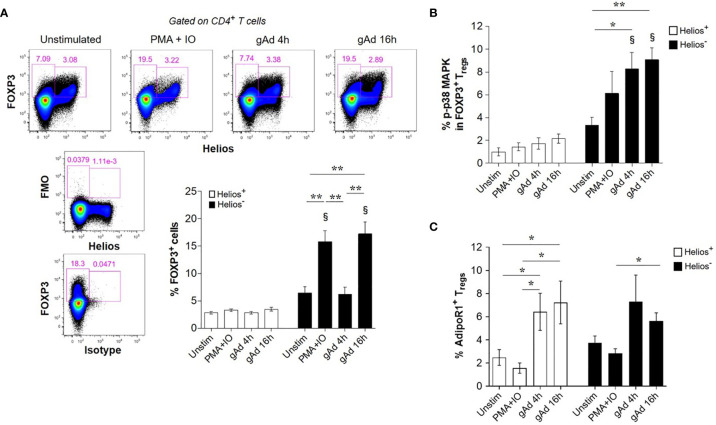
gAd induces FOXP3 in CD4^+^ T cells, p38 MAPK activation in Helios^-^ cells and AdipoR1 expression in Tregs. CD4^+^ T cells were cultured in the presence of gAd and then analyzed by flow cytometry. **(A)** Representative FACS plots show FOXP3 and Helios expression in CD4^+^ T cells treated with gAd (4 h or 16 h) or PMA/ionomycin (4 h). The graph summarizes the frequencies of FOXP3^+^ Helios^+^ Tregs or FOXP3^+^ Helios^-^ Tregs following treatments. Analysis of FOXP3 and Helios was performed in CD4^+^ T cells. **(B)** Frequency of phosphorylated p38 (p-p38) MAPK positive cells within FOXP3^+^ Helios^+^ Tregs and FOXP3^+^ Helios^-^ Tregs. **(C)** Frequency of AdipoR1 in Helios^+^ and Helios^-^ FOXP3^+^ Tregs upon treatment with gAd or PMA/IO. Data are means ± SEMs of n= 4-6 blood donors. Student’s t-test, §P<0.01 in comparisons between Helios^+^ and Helios^-^ cells with their respective treatment or for one-to-one comparisons **(C)**. One-way ANOVA with Tukey’s multiple comparisons test, *P< 0.05 and **P< 0.01.

We next investigated whether gAd differentially affects FOXP3 expression in Helios^-^ and Helios^+^ cells. Neither gAd nor PMA/ionomycin treatments affected the expression of FOXP3 in Helios^+^ cells (**Figure 4A**). In contrast, treatment either with PMA/ionomycin for 4 h or gAd for 16 h, but not for 4 h, markedly increased the expression of FOXP3 in Helios^-^ cells (**Figure 4A**). Because the conversion of conventional CD4^+^ T cells (FOXP3^-^ cells) into FOXP3^+^ Tregs is mediated by p38 MAP kinase (MAPK) signalling pathway (43), we next investigated whether gAd treatment activates p38 MAPK in FOXP3^+^ Tregs. As shown in **Figure 4B**, gAd treatment significantly induced p38 MAPK phosphorylation in FOXP3^+^ Helios^-^ Tregs but not in FOXP3^+^ Helios^+^ Tregs (**Figure 4B**). Furthermore, we investigated whether gAd treatment could affect the expression of AdipoR1 in Tregs. Interestingly, gAd treatment for 4 and 16 h increased the frequency of AdipoR1 in FOXP3^+^ Helios^+^ Tregs compared to unstimulated and PMA/ionomycin treated cells (**Figure 4C**). Moreover, gAd treatment for 16 h increased the frequency of AdipoR1 in FOXP3^+^ Helios^-^ Tregs compared to PMA/ionomycin treatment. Together, these results suggest that gAd affects the biology of Tregs by inducing FOXP3 expression and activation of p38 MAPK in Helios^-^ Tregs, but also by upregulating the expression of AdipoR1.

### AdipoRon Induces FOXP3 Expression and IL-10 Release in Helios^-^ AdipoR1^+^ Tregs

**Figure 5:**
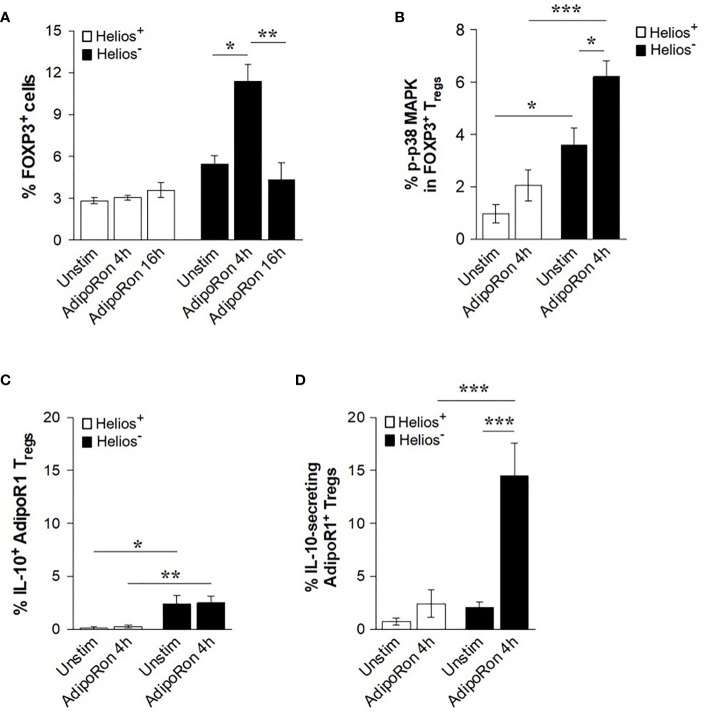
AdipoRon induces FOXP3 expression and IL-10 release from Helios^-^ AdipoR1^+^ Tregs. CD4^+^ T cells were cultured in the presence of AdipoRon and then analyzed by flow cytometry. **(A)** Frequency of FOXP3^+^ Helios^+^ Tregs or FOXP3^+^ Helios^-^ Tregs. **(B)** Frequency of phosphorylated p38 (p-p38) MAPK cells within FOXP3^+^ Helios^+^ Tregs and FOXP3^+^ Helios^-^ Tregs. **(C)** Frequency of intracellular IL-10^+^ cells in AdipoR1^+^ Tregs. **(D)** Frequency of IL-10-secreting AdipoR1^+^ Tregs. The analysis of intracellular IL-10^+^ cells or IL-10-secreting cells was performed in CD4^+^ FOXP3^+^ Helios^+^ AdipoR1^+^ Tregs and CD4^+^ FOXP3^+^ Helios^-^ AdipoR1^+^ Tregs. Data are means ± SEM of n=4-6 blood donors. One-way ANOVA with Tukey’s multiple comparisons test, and Student’s t-test for one-to-one comparisons,*P< 0.05, **P< 0.01, and ***P< 0.001.

To further validate gAd effects on the function of Treg subsets, CD4^+^ T cells were treated with AdipoRon, a synthetic agonist of adiponectin receptors (44). We found that while AdipoRon did not affect the frequency of FOXP3 in Helios^+^ cells, it significantly increased the frequency of FOXP3 in Helios^-^ cells at 4 h, but not at 16 h of treatment (**Figure 5A**). Similarly, when p38 MAPK activation was assessed after 4 h of treatment with AdipoRon, we found that it induced p38 MAPK phosphorylation in Helios^-^ Tregs but not in Helios^+^ Tregs (**Figure 5B**). We next examined whether AdipoRon induces the IL-10 production in AdipoR1^+^ Tregs. AdipoRon did not affect the intracellular levels of IL-10 in either Helios^+^ or Helios^-^ AdipoR1^+^ Tregs (**Figure 5C**). Interestingly, AdipoRon significantly increased the frequency of IL-10-secreting Helios^-^ AdipoR1^+^ Tregs as compared with both unstimulated cells and Helios^+^ AdipoR1^+^ Tregs (**Figure 5D**). Together, these findings indicate that both gAd and AdipoRon activate, albeit at different time points, Helios^-^ AdipoR1^+^ Tregs by increasing the secretion of IL-10, the expression of FOXP3 and the phosphorylation of p38 MAPK.

### gAd Induces the Production of IL-10 in AdipoR1^+^ Tregs in a T2 Cytokine Milieu

**Figure 6:**
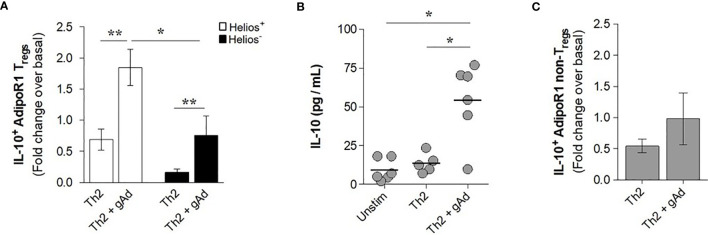
gAd induces the production of IL-10 in AdipoR1^+^ Tregs in a T2 cytokine milieu. CD4^+^ T cells were cultured under a T2 cytokine milieu in the presence or absence of gAd for 5 days. **(A)** Intracellular IL-10^+^ AdipoR1^+^ Tregs were assessed by flow cytometry and results were expressed as fold increases in the frequency of positive cells over basal (unstimulated cells). **(B)** IL-10 levels were determined in culture supernatants by ELISA. **(C)** Intracellular IL-10^+^ AdipoR1^+^ non-Tregs (CD4^+^ FOXP3^-^ cells) were assessed by flow cytometry and results were expressed as fold increases in the frequency of positive cells over basal (unstimulated cells). Data are means ± SEM of n=5-6 blood donors. One-way ANOVA with Tukey’s multiple comparisons test, and Student’s t-test for one-to-one comparisons, *P< 0.05 and **P< 0.01.

We next investigated whether gAd ability to induce IL-10 production is affected under T2 inflammatory environment. To this end, CD4^+^ T cells were cultured for 5 days in T2 cytokine milieu in presence or absence of gAd and the frequency of IL-10^+^ cells was assessed in Helios^+^ and Helios^-^ AdipoR1^+^ Tregs. Strikingly, we found that under T2 inflammatory conditions, gAd induced a significant increase in the intracellular expression of IL-10 in both Helios^+^ and Helios^-^ AdipoR1^+^ Tregs as compared with cells under T2 conditions alone (**Figure 6A**). The induction of IL-10 by gAd under T2 conditions was significantly higher in Helios^+^ AdipoR1^+^ Tregs as compared with Helios^-^ AdipoR1^+^ Tregs (**Figure 6A**). Similarly, IL-10 levels were significantly augmented in supernatants of CD4^+^ T cells cultured under T2 conditions in the presence of gAd as compared with T2 conditions alone or unstimulated cells (**Figure 6B**). Together, these findings suggest that T2 inflammation amplifies adiponectin effect on Helios^+^ Tregs functions. To examine whether AdipoR1^+^ conventional CD4^+^ T cells contribute to IL-10 accumulation in response to gAd, we assessed intracellular IL-10 expression in AdipoR1^+^ non-Tregs (CD4^+^ FOXP3^-^ cells). As shown in **Figure 6C**, we found that the addition of gAd under T2 cytokine milieu did not affect the frequency of IL-10^+^ AdipoR1^+^ non-Tregs (**Figure 6C**). Together, these findings indicate that under T2 inflammation, Helios^+^ AdipoR1^+^ Tregs are the primary source of IL-10 in response to gAd.

## Discussion

In this study, we report that human Tregs express the AdipoR1 and produce more IL-10 than AdipoR1^-^ Tregs. Moreover, *ex vivo* study of Treg subpopulations, defined by Helios expression, revealed that gAd isoform induces IL-10 secretion from Helios^-^ AdipoR1^+^ Tregs, while under T2 inflammation Helios^+^ AdipoR1^+^ Tregs were the primary source of IL-10. Importantly, we show that gAd or the synthetic agonist of AdipoRs, AdipoRon, increase the expression of FOXP3 in Helios^-^ CD4^+^ T cells. All above findings reveal a novel mechanism for the anti-inflammatory effect of adiponectin in human Tregs further suggesting adiponectin/AdipoR1 axis as a suitable therapeutic option for the treatment of inflammatory conditions.

AdipoRs are expressed in human immune cells, where CD4^+^ T cells preferentially express the AdipoR1 (37, 38). We previously showed that CD4^+^ FOXP3^+^ Tregs express AdipoR1 in mouse tissues (15, 45); however, whether human Tregs express AdipoR1 has not been explored. In the present study, we report that both Helios^+^ and Helios^-^ Tregs express AdipoR1. Because adiponectin exerts anti-inflammatory effects on multiple immune cells, we speculate that AdipoR1 may also mediate such effects in Tregs. Our data show that AdipoR1^+^ Tregs produce significantly higher levels of IL-10 compared to AdipoR1^-^ Tregs. An interesting finding in our study was that subpopulations of Tregs, determined by expression of the transcription factor Helios, differentially expressed AdipoR1 and produced IL-10. Although Helios has been proposed as a marker to discriminate tTregs (FOXP3^+^ Helios^+^ Tregs) from pTregs (FOXP3^+^ Helios^-^ Tregs) in human and mouse (6, 7), it has also been reported that tTregs possess a mixture of Helios^+^ and Helios^-^ cells (46). Furthermore, pTregs generated *in vitro* and *in vivo* acquire some levels of Helios depending on stimulation conditions (47–49). Our findings confirm previous data showing that Helios^– ^Tregs produce significantly more IL-10 compared to Helios^+^ Tregs (6, 39, 40, 46). Interestingly, we also found a subpopulation of Helios^+^ Tregs that express AdipoR1^+^ and produce IL-10. Recent studies show that Tregs are functionally heterogeneous and adapt their phenotype to different environments to suppress cell/tissue-specific responses (50, 51). Accordingly, our group previously showed that AdipoR1 expression in Helios^+^ Tregs negatively correlated with epididymal fat in a mouse model of diet-induced obesity (DIO) (15). More recently, we showed that severe obesity augmented the frequency of AdipoR1^+^ Tregs in lungs, suggesting that adipose tissue-related Tregs might reach distant tissues to control systemic inflammation (45). In our current study, we demonstrate that human Tregs also express the AdipoR1 and produce IL-10. Interestingly, AdipoR1^+^ Tregs express a much higher level of IL-10 than AdipoR1^-^ Tregs, which suggests that adiponectin might have a role in the IL-10-mediated regulatory functions of this subset of Tregs.

Because adiponectin induces the production of IL-10 in human monocytes (32), it was plausible that AdipoR1^+^ Tregs treated with adiponectin produce IL-10. However, we found that levels of intracellular IL-10 in AdipoR1^+^ Tregs were not affected by treatment with gAd. Evidence obtained from mouse studies shows that adipose tissue Tregs express up to 136-fold increase of IL-10 transcripts compared to lymph node Tregs (14). We speculate that AdipoR1^+^ Tregs might also accumulate IL-10 and adiponectin would induce its secretion. Indeed, the IL-10 secretion assay showed that gAd induced the rapid secretion of IL-10 (4 h) in Helios^-^ AdipoR1^+^ Tregs, but also at later time point (16 h). In line with this, studies performed in macrophages treated with gAd showed that maximal accumulation of IL-10 mRNA occurred at 5 h while a significant increase of IL-10 in cell supernatants was seen at 2 h (52). Furthermore, Kumada and colleagues demonstrated that IL-10 mRNA levels started to increase at 6 h of adiponectin treatment and continued elevated at 48 h while IL-10 protein was detected in culture supernatants within 24 h (33). In our study, a major limitation to trace the IL-10 production from mRNA to secreted protein in AdipoR1^+^ Tregs is the absence of known surface markers to discriminate between Helios^+^ and Helios^-^ Tregs. However, although further studies are needed to address the molecular mechanisms that regulate the functions of AdipoR1^+^ Tregs, our data suggest that AdipoR1 expression distinguish a subset of IL-10-producing Tregs, where gAd might selectively induce the rapid secretion of IL-10 in Helios^-^ AdipoR1^+^ Tregs.

A major finding in our study is that gAd induces the expression of FOXP3. Interestingly, studies performed in mouse cells demonstrated that dendritic cells differentiated in the presence of adiponectin, and subsequently co-cultured with CD4^+^ T cells, were able to induce FOXP3^+^ Tregs compared with untreated dendritic cells (34). Moreover, a recent study conducted in a mouse model of autoimmune encephalomyelitis showed that treatment with gAd reduced the expression of inflammatory markers and increased the frequency of FOXP3^+^ Tregs (53). To our knowledge, this is the first study showing that gAd directly influences the biology of human Tregs. Cheng and colleagues found that full-length adiponectin promoted Th1 lineage and did not alter the expression of FOXP3 (38). These data suggest that adiponectin isoforms differentially affect the biology of CD4^+^ T cells. A striking result in our study was that the induction of FOXP3 occurred in Helios^-^ but not in Helios^+^ cells, which is in line with the hypothesis that Helios^-^ cells correspond to the phenotype of pTregs. Although we cannot determine the origin of AdipoR1^+^ Tregs only based on their differential expression of Helios, it is possible that the expression of Helios in these subpopulations of Tregs correlates with a more stable FOXP3 expression. Interestingly, we show that gAd induces the activation of p38 MAPK in Helios^−^ Tregs but not in Helios^+^ Tregs, which is consistent with a previous study showing that p38 MAPK signalling is required for the generation of pTreg (43). Thus, it is plausible that Helios^-^ Tregs with a marked activation of p38 MAPK correspond to pTregs. Furthermore, our data also reveal that gAd upregulates the expression of AdipoR1 in Tregs. Importantly, this effect was not due to FOXP3 induction upon gAd treatment as AdipoR1 expression was elevated in both Helios^+^ and Helios^-^ Tregs whereas FOXP3 induction was observed only in Helios^-^ cells. Accordingly, it has been shown that the levels of adiponectin and AdipoRs are directly related. For instance, adiponectin levels and AdipoR1/R2 expression levels are both decreased in obesity (54, 55). Additional studies performed in mice showed that deficiency of AdipoR1, but not of AdipoR2, resulted in an obese phenotype (56). In line with this, we previously showed that AdipoR1 expression in Tregs is reduced in obese mice (15). Hence, it is possible that gAd/AdipoR1 axis may exert a protective role against inflammatory conditions, such as obesity, where the upregulation of AdipoR1 through its own ligand may increase the sensitivity of Tregs to adiponectin, which in turn promotes IL-10 production.

Our data show that AdipoRon induces the expression of FOXP3 at an early time point (4 h) but not at a later time point (16 h). In contrast, gAd induces the expression of FOXP3 at 16 h of stimulation but not at 4 h of stimulation. This apparent discrepancy may be due to the nature of these ligands. While AdipoRon is a synthetic chemical compound and the most potent agonist for AdipoRs (44), gAd is the natural/native ligand for AdipoR1 (24, 26). Although we do not exclude the possibility that the early effects of AdipoRon on the induction of Tregs could be mediated by both AdipoRs, previous studies have shown that AdipoR1 primarily activates the AMPK and p38 MAPK pathways, whereas AdipoR2 mainly acts through COX-2 and PPARα/γ pathways (42). Since p38 MAPK pathway signal has been shown to be involved in the induction of FOXP3 and IL-10 production (42, 43), pathways previously shown to be activated by AdipoR1, we believe that the effect of AdipoRon on the induction of FOXP3 is mediated by AdipoR1; however, further studies are needed to validate this hypothesis. Because AdipoRon induced FOXP3 expression at early time points, we investigated its effects following 4 h of treatment. Similar to the results obtained with gAd, AdipoRon induced p38 MAPK activation in Helios^-^ cells and IL-10 secretion by Helios^-^ AdipoR1^+^ Tregs. AdipoRon is the most well studied synthetic agonist of AdipoRs (44, 57). It possess anti-diabetic and anti-atherogenic effects (44, 58); however, the direct effects of AdipoRon on immune cells are not completely defined. A study performed in muscle cells obtained from a mouse model of Duchenne muscular dystrophy revealed that the treatment with AdipoRon augmented the expression of IL-10 mRNA and protein as compared to untreated animals (59). Moreover, this study demonstrated that AdipoRon significantly reduced pro-inflammatory cytokines, indicating that AdipoRon might be beneficial in the treatment of inflammatory diseases (59). Interestingly, anti-inflammatory effects of AdipoRon were abolished by silencing the AdipoR1 gene, suggesting that such functions were mediated by AdipoR1 (59). In our study, we did not determine whether AdipoRon induced the IL-10 production in Helios^-^ AdipoR1^+^ Tregs *via* AdipoR1 or AdipoR2. However, AdipoR1 has been strongly associated with the anti-inflammatory effects of adiponectin, particularly the gAd isoform (24, 59, 60). Here, we suggest the AdipoR1 as a potential target for specific agonists that might modulate the anti-inflammatory functions of Tregs.

We previously showed that DIO mice under allergic asthma model showed a reduced frequency of Helios^-^ AdipoR1^+^ Tregs in the lungs compared to DIO non-allergic mice (45). In the current study, we investigated whether allergic T2 inflammatory environment may affect human AdipoR1^+^ Tregs. Strikingly, we found that under T2 inflammatory conditions gAd induced IL-10 production not only in Helios^-^ AdipoR1^+^ Tregs but also in Helios^+^ AdipoR1^+^ Tregs, indicating that this latter subpopulation might respond to gAd by producing IL-10 under specific inflammatory conditions. The effect of gAd/AdipoR1 axis on anti-inflammatory responses, including the induction of IL-10, has been observed in several cell types (52, 60, 61). Mandal and colleagues reported that IL-10 is required for gAd to suppress LPS-induced TNFα expression in Kupffer cells. Notably, this effect was abolished by knocking down AdipoR1 but not AdipoR2 (61). In mouse macrophages, gAd inhibited NF-κB signaling through AdipoR1 (62), while full-length adiponectin acted *via* AdipoR2 to shift macrophages toward a M2 phenotype utilizing an IL-10 independent mechanism (63). Our data suggest that under a T2 inflammatory environment, gAd is able to induce IL-10 production in AdipoR1^+^ Tregs. Although gAd is not typically detected in the circulation, the full-length adiponectin is cleaved in specific tissues or at sites of inflammation (23, 64), suggesting that the functions of gAd are mediated by inflammatory signals. Because of the robust association between reduced adiponectin levels and obesity-related diseases (65, 66), it is of significant interest to understand the molecular mechanisms that regulate the adiponectin/AdipoRs functions. Accordingly, our data show that upon gAd or AdipoRon treatment, CD4^+^ T cells acquire FOXP3 expression. Therefore, the development of new compounds that mimic or enhance adiponectin actions in an isoform-specific manner would be an attractive therapeutic option to ameliorate metabolic or inflammatory disorders. Particularly, our findings reveal a novel mechanism through which gAd and AdipoRon might induce anti-inflammatory effects by upregulating Tregs and IL-10 release.

Here, we reported for first time the existence of IL-10-producing AdipoR1^+^ Tregs in human; however, further studies are still needed to elucidate the role of this subpopulation in *in vivo* inflammatory conditions. In addition, our study has some limitations. For example, our samples were anonymized where we do not have access to any demographic information, such as gender or age, which may influence the expression and the function of AdipoR1/adiponectin axis. Also, our study used a small number of samples. However, the main objective of this study was to explore the expression and the function of AdipoR1 in human Tregs in response to adiponectin. We believe that due to our straightforward approach, we manage to address such goals despite this limitation. However, we recognize that the above limitations need to be addressed in future studies by using bigger sample size and additional experimental approaches.

**Figure 7:**
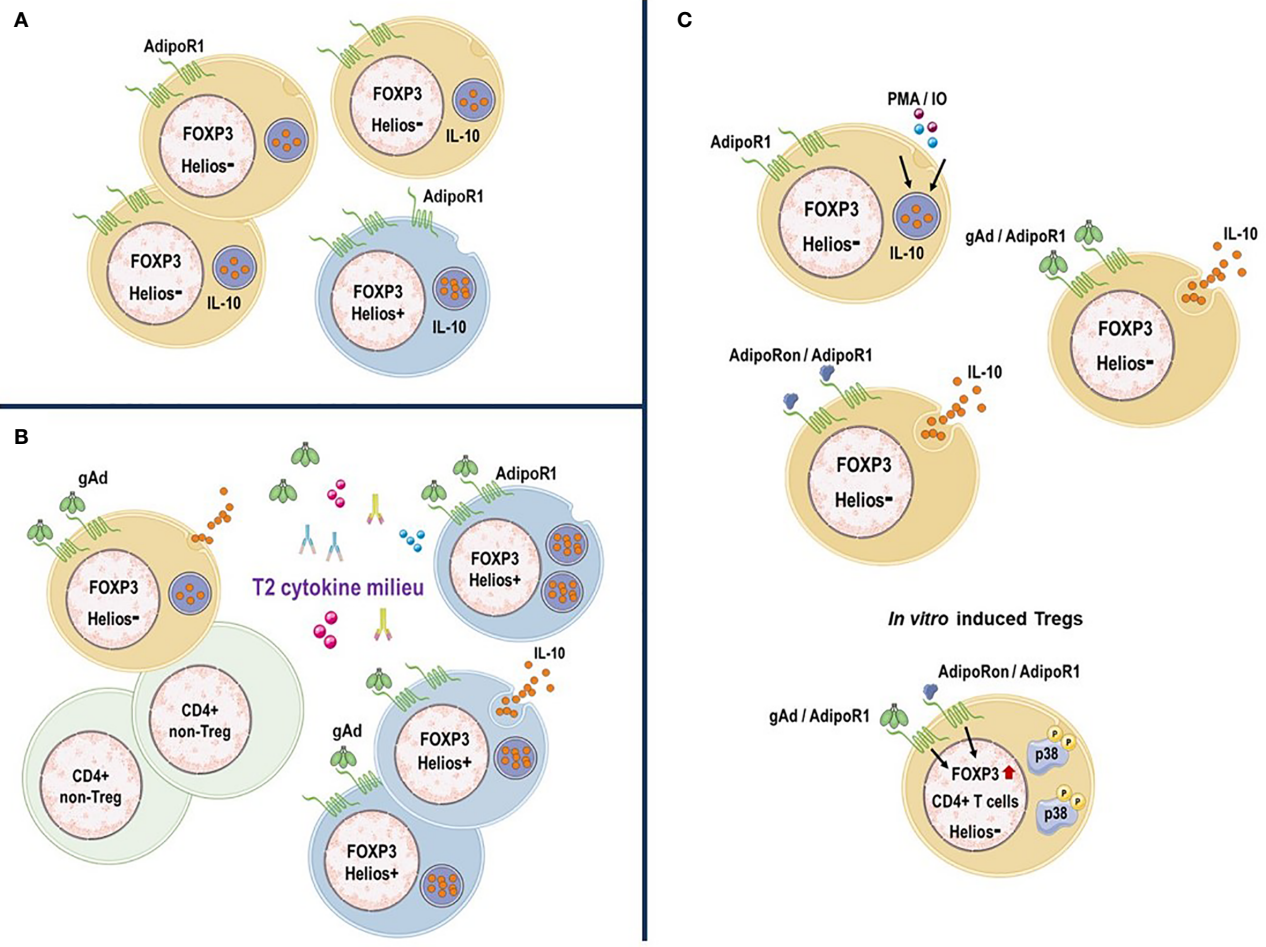
AdipoR1 expression distinguish a subpopulation of IL-10-producing Tregs. **(A)** AdipoR1^+^ Tregs in human peripheral blood. Human circulating Tregs express AdipoR1 and produce IL-10. While there is a high frequency of AdipoR1^+^ cells that produce IL-10 within Helios^-^ Tregs, AdipoR1 and IL-10 expression (MFI) is upregulated within Helios^+^ Tregs. **(B)** *In vitro* activated AdipoR1^+^ Tregs. gAd or AdipoRon induce IL-10 release in Helios^-^ AdipoR1^+^ Tregs. gAd or AdipoRon promote the generation of FOXP3^+^ Helios^-^ Tregs likely through activation of p38 MAPK. **(C)** AdipoR1^+^ Tregs under T2 cytokine conditions. T2 inflammatory environment amplifies the effect of gAd on IL-10 production by AdipoR1^+^ Tregs, where Helios^+^ AdipoR1^+^ Tregs are the primary source of IL-10. *Figure was created using templates from Servier Medical Art, which are licensed under a Creative Commons Attribution 3.0 Unported License (* http://smart.servier.com/*)*.

In conclusion, human circulating AdipoR1^+^ Treg subpopulations differ in their regulatory properties and can be subdivided based on their Helios expression. Our data suggest that under inflammatory conditions the gAd/AdipoR1 axis might play a critical role in regulating the functions of Helios^+^ and Helios^-^ AdipoR1^+^ Tregs where T2 inflammation amplifies the effect of gAd on IL-10 production by Helios^+^ AdipoR1^+^ Tregs (**Figure 7**). Further studies are still needed to determine the molecular mechanisms underlying the effect of gAd on AdipoR1^+^ Tregs; however, the induction of AdipoR1 and IL-10 production in Tregs suggests a potential novel strategy to attenuate inflammatory responses.

## Data Availability Statement

The data generated in this study are available on request to the corresponding author.

## Ethics Statement

Peripheral blood was collected from healthy volunteers who gave their oral informed consent under the ethical approval granted by the regional Ethical Approval Committee (no 593-08). The fresh buffy coats were obtained from healthy blood donors (Blood Donor Center, Sahlgrenska University Hospital, Gothenburg). All samples were completely anonymized without access to any data about the donors.

## Author Contributions 

PR-R and AB conceived the study, designed the experiments, analyzed data, and wrote the manuscript. PR-R and CM performed the experiments. CM, MR, and OT contributed to data interpretation and provided critical comments to the manuscript. All authors reviewed and commented on the manuscript. All authors contributed to the article and approved the submitted version.

## Funding

This study was supported by grants from Herman Krefting Foundation, the Swedish Heart Lung Foundation (Ref. 20120565, 20170747, 20180219, 20180220, 20200619), and the Swedish Cancer and Allergy Foundation (AB); National Institutes of Health grants R01HL111541 (OT). PR-R was supported by Consejo Nacional de Ciencia y Tecnología (CONACyT), México (Ref. 232150/261200).

## Conflict of Interest

The authors declare that the research was conducted in the absence of any commercial or financial relationships that could be construed as a potential conflict of interest.
